# Protective effect of alpha-mangostin against oxidative stress induced-retinal cell death

**DOI:** 10.1038/srep21018

**Published:** 2016-02-18

**Authors:** Yuan Fang, Tu Su, Xiaorong Qiu, Pingan Mao, Yidan Xu, Zizhong Hu, Yi Zhang, Xinhua Zheng, Ping Xie, Qinghuai Liu

**Affiliations:** 1 Department of Ophthalmology, The First Affiliated Hospital of Nanjing Medical University, Nanjing, Jiangsu 210029, China

## Abstract

It is known that oxidative stress plays a pivotal role in age-related macular degeneration (AMD) pathogenesis. Alpha-mangostin is the main xanthone purified from mangosteen known as anti-oxidative properties. The aim of the study was to test the protective effect of alpha-mangostin against oxidative stress both in retina of light-damaged mice model and in hydrogen peroxide (H_2_O_2_)-stressed RPE cells. We observed that alpha-mangostin significantly inhibited light-induced degeneration of photoreceptors and 200 μM H_2_O_2_-induced apoptosis of RPE cells. 200 μM H_2_O_2_-induced generation of reactive oxygen species (ROS) and light-induced generation of malondialdehyde (MDA) were suppressed by alpha-mangostin. Alpha-mangostin stimulation resulted in an increase of superoxide dismutase (SOD) activity, glutathione peroxidase (GPX) activity and glutathione (GSH) content both *in vivo* and *vitro.* Furthermore, the mechanism of retinal protection against oxidative stress by alpha-mangostin involves accumulation and the nuclear translocation of the NF-E2-related factor (Nrf2) along with up-regulation the expression of heme oxygenas-1 (HO-1). Meanwhile, alpha-mangostin can activate the expression of PKC-δ and down-regulate the expression of mitogen-activated protein kinases (MAPKs), including ERK1/2, JNK, P38. The results suggest that alpha-mangostin could be a new approach to suspend the onset and development of AMD.

Age-related macular degeneration (AMD) is the leading cause of blindness in the elderly population in the western countries^1^, which affects the macular and as a consequence results in central vision loss. AMD is characterized by including accumulation of extracellular deposits (drusen) in the macula, thickening of Bruch’s membrane, degeneration of retinal pigment epithelial (RPE) cells and photoreceptors, and even abnormal neovascularization (angiogenesis) growing into the central region of the retina^2^.

Although the pathogenesis of AMD is still not well understood, oxidative stress is believed to accelerate the process of aging and contributes to the development of AMD^3^. The retina, a tissue with high levels of various lipid compounds^4^ in the environment with high oxygen tension^5^, along with high levels of light exposure, is particularly predisposed to produce reactive oxygen species (ROS). Mitochondria represent a major source of endogenous ROS in most cells of the retina^6^. ROS in turn damages mitochondria and lipids, leading to modification of retinal function. In recent studies, many evidences have suggested that light exposure can be involved in the progress of AMD^7^. Epidemiological studies on a population with similar lifestyle have indicated a positive correlation between AMD and the accumulated exposure to solar radiation^8^,^9^. In laboratory^10^,^11^, retina damages after light exposure in animal models which are generally similar to those seen in patients with AMD is induced by photochemical and photo-oxidative mechanisms. Furthermore, studies^12^,^13^ showed that the administration of antioxidants targeting ROS generation and oxidative damage can protect retina from light damage and play a therapeutic role in animal model of AMD. Therefore, the anti-oxidant strategy plays an important role in the treatment of AMD.

Alpha-mangostin, a yellow color matter, is the major xanthone purified from mangosteen. Alpha-mangostin has health promoting benefits including anti-bacterial^14^, anti-inflammatory^15^, anti-oxidant^16^, anti-cancer^17^ and cardio protective^18^ activities. Researches showed that alpha-mangostin can inhibit the oxidation of low-density lipoprotein *in vitro*^19^ and scavenge singlet oxygen, superoxide anion and peroxynitrite anion^20^. This antioxidant as a free radical scavenger ameliorates the neuronal death induced by 3-nitropropionic acid (3-NP)^21^. Treatment of alpha-mangostin also provided a neuroprotective effect on cerebellar granule neurons (CGNs) which was associated with the induction of heme oxygenase-1 (HO-1)^22^. Although alpha-mangostin has showed several antioxidant properties, the effects on retina health have not been clarified yet.

In our study, we investigated the effects of administration with alpha-mangostin in animal models of light–induced retina degeneration and the underlying mechanisms were discussed.

## Results

### *In vivo*

#### Alpha-mangostin suppressed light-Induced retina dysfunction

**Figure 1:**
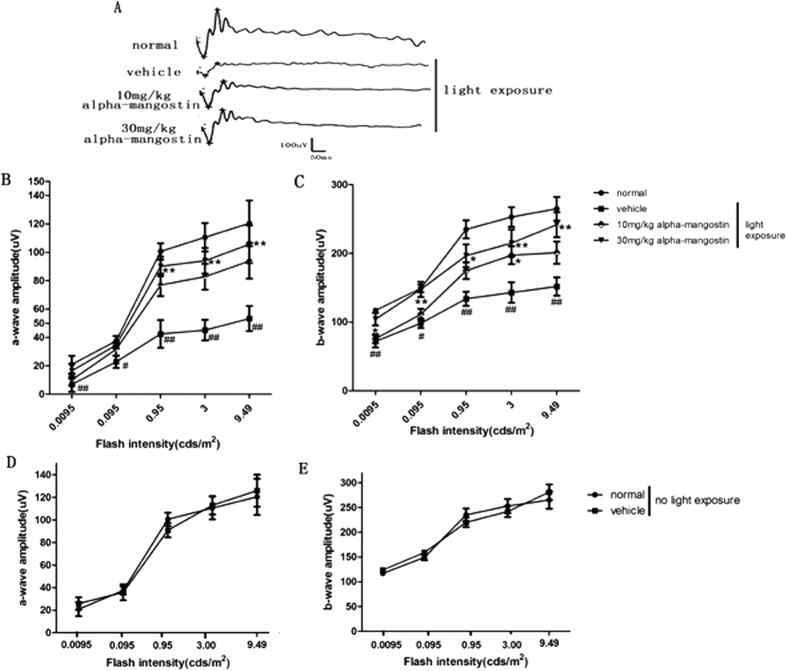
Effects of alpha-mangostin on light-induced retinal dysfunction in mice at 3days after light exposure (5000lux). Stimulus flashes were used from 0.0095 to 9. 49 cds/m^2^. (**A**) Scotopic ERG recorded at the flash intensity of 3.00 cds/m^2^. Quantification of scotopic-a wave amplitudes (**B**) and scotopic-b wave amplitudes (**C**) for normal mice, vehicle mice with light exposure,10 mg/kg alpha-mangostin mice with light exposure and 30 mg/kg alpha-mangostin mice with light exposure. Quantification of scotopic-a wave amplitudes (**D**) and scotopic-b wave amplitudes (**E**) for vehicle mice and 30 mg/kg alpha-mangostin mice without light exposure. Data are expressed as the mean ± SD, n = 9. Significant differences were calculated using one-way ANOVA followed by Tukey’s multiple comparison test. *P < 0.05, **P < 0.01 vs. the vehicle mice with light exposure, ^#^P < 0.05, ^##^P < 0.01 vs. the normal mice.

We investigated the role of alpha-mangostin on light-induced retinal dysfunction of mice by ERG analysis 3 days after light exposure. The vehicle-treated mice with light exposure showed significant reduction in the amplitudes of a- and b-waves compared with the normal mice, the amplitudes of a-wave and b-wave decreased by 56% and 43%, respectively, at the flash intensity of 3.00 cds/m^2^. Treatment with higher-dose (30 mg/kg) of alpha-mangostin before light exposure could inhibit the reduction of these amplitudes effectively at the maximum flash intensity by 43% and 34%, respectively. Alpha-mangostin at a dose of 10 mg/kg body weight inhibited the reduction of these amplitudes by 34% and 19%, respectively. The differences between lower-dose (10 mg/kg) mice with light exposure and vehicle-treated mice with light exposure was not statistically significant (P > 0.05). Additionally, it should be noted that without excessive light exposure the difference of amplitudes of a-wave and b-wave were indistinguishable between the vehicle-treated mice and higher-dose mice. The above results indicated that treatment with alpha-mangsotin at the dose of 30 mg/kg body weight prevented loss of photoreceptor function to a greater extent in the presence of light damage than the dose of 10 mg/kg (Fig. 1). Thus, we chose alpha-mangostin at 30 mg/kg body weight *in vivo* for the following studies.

#### Alpha-mangostin suppressed light-induced ONL thinning

**Figure 2:**
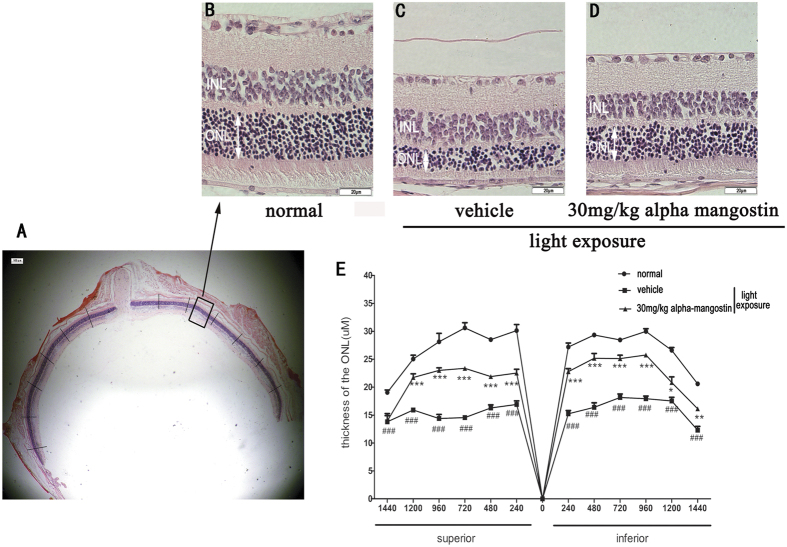
Effects of alpha-mangostin on light-induced ONL thinning in mice retinal at 3days after light exposure (5000lux). ONL, outer nuclear layer; INL, inner nuclear layer. (**A**) The area of representative retinal image was indicated by a rectangle frame. (**B**) Higher magnification of one representative retinal image of H&E staining in the normal mice between 480 μM and 720 μM from the optic nerve in the superior area. (**C**) The same magnfication image of retina in vehicle mice with light exposure. (**D**) Retinal image in 30 mg/kg alpha-mangostin mice with light exposure. (**E**) Data are expressed as the mean ± SD, n = 5. Significant differences were calculated using one-way ANOVA followed by Tukey’s multiple comparison test. *P < 0.05, **P < 0.01, and ***P < 0.001 vs. the vehicle mice with light exposure, ^#^P < 0.05, ^##^P < 0.01, ^###^P < 0.001 vs. the normal mice.

To examine the effect of alpha-mangostin on light-induced histological damage to the retina, we measured the thickness of the ONL and compared the results of each group. Figure 2A–C showed the representative retinal images between 480 μm and 720 μm from the optic nerve in the superior area. In normal mice, the mean thickness of ONL in superior and inferior hemiretina was 26.91 ± 4.32 μm and 26.99 ± 3.80 μm, respectively. 3 days after light exposure, we observed a remarkable decrease in ONL thickness both in the superior and the inferior hemiretina of the vehicle-treated mice. And the mean ONL thickness was reduced by 40% (15.31 ± 1.23, P < 0.001) in the inferior retina and 44% (16.46 ± 2.42, P < 0.001) in the superior retina versus the normal mice. On the other hand, there was only 21% (22.51 ± 0.70, P < 0.01) and 16% (22.63 ± 3.68, P < 0.01) reduction in mean ONL thickness of inferior and the superior hemiretina, respectively, in mice treated with alpha-mangostin (30 mg/kg). Administration of alpha-mangostin (30 mg/kg) to light-exposed mice significantly suppressed the thinning of ONL thickness induced by light exposure. Without light exposure, alpha-mangostin did not affect ONL thickness (data not shown). Overall, the results revealed that alpha-mangostin supplementation protects the function and structure of retina from light-induced damage (Fig. 2E).

#### Alpha-mangostin suppressed light-Induced cell apoptosis in Retina

**Figure 3:**
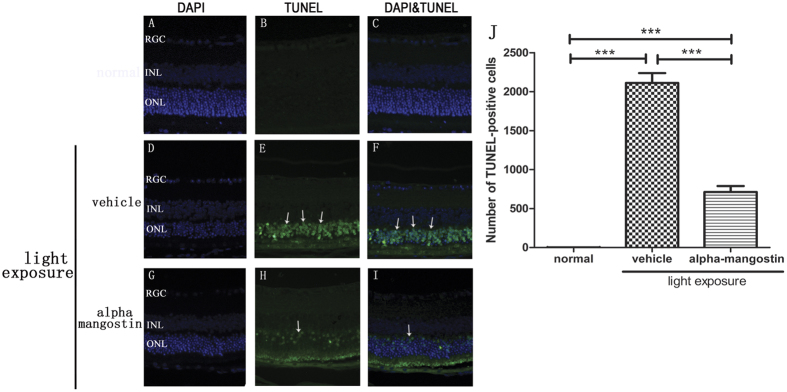
Effects of alpha-mangostin on light-induced expression of TUNEL-positive cells in mice retina at 3 days after light exposure (5000lux). (**A**–**C**) Fluorescence photomicrographs of normal mice. (**D**–**F**) Fluorescence photomicrographs of vehicle mice with light exposure. (**G**–**I**) Fluorescence photomicrographs of alpha-mangostin (30 mg/kg) mice with light exposure. Cell nuclei (blue) were counterstained with DAPI. TUNEL-positive cells (green, arrow) mainly expressed in the outer nuclear layer (ONL). (**J**) Quantitative analysis of the number of TUNEL-positive cells in the outer nuclear layer at 3 days after light exposure. Data are expressed as the mean ± SD, n = 5. Significant differences were calculated using one-way ANOVA followed by Tukey’s multiple comparison test. ***P < 0.001.

Apoptotic cells were primarily detected in the outer nuclear layer of the retina. TUNEL staining was examined 24 hours after light exposure and assessed the number of TUNEL-positive cells. It showed the protective effect of alpha-mangostin on light-induced cell apoptosis. In normal mice, there was no TUNEL-positive cell (1.75 ± 1.71) observed in any area of retina. Conversely, exposure to light increased the TUNEL-positive cells markedly in the vehicle-treated retina (2112.50 ± 252.90, P < 0.0001). Whereas, quantitative analysis suggested that administration of alpha-mangostin (30 mg/kg) significantly reduced the number of TUNEL-positive cells induced by light exposure, and the reduction was approximately 65% (712.00 ± 154.84, P < 0.0001) (Fig. 3).

#### Nrf2 expressed in the ONL in frozen retinal sections

**Figure 4:**
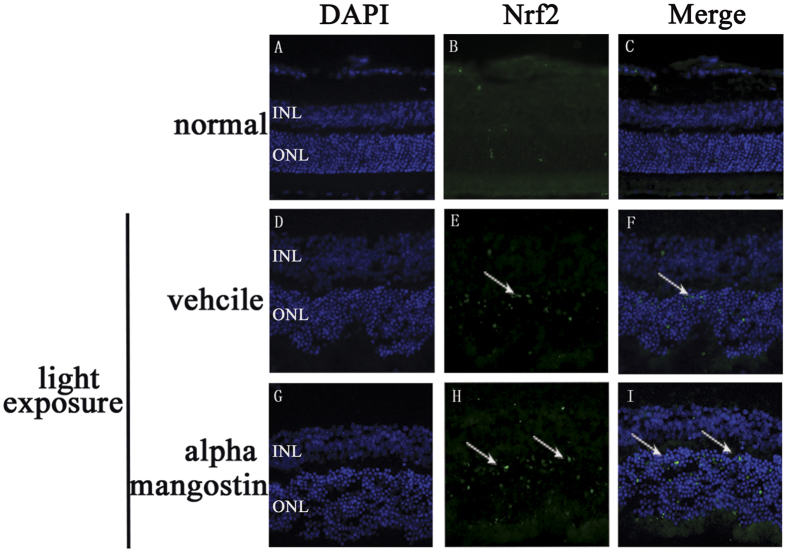
Effects of alpha-mangostin on the expression of Nrf2 in outer nuclear layer (ONL) in mice retina at 3 days after light exposure (5000lux). Nrf2 immunoactivity (green, arrow) was detected in outer nuclear layer (ONL). Cell nuclei (blue) were counterstained with DAPI. (**A**–**C**) Fluorescence photomicrographs of normal mice. (**D**–**F**) Fluorescence photomicrographs of vehicle mice with light exposure. (**G**–**I**) Fluorescence photomicrographs of alpha-mangostin (30 mg/kg) mice with light exposure.

Under oxidative or chemical stress, the activated Nrf2 translocates into the nucleus, and interacts with ARE, then antioxidant genes are activated to attenuate cellular oxidative stress. We did immunofluorescence analysis of retinal tissues to determine whether alpha-mangostin pretreatment could cause Nrf2 up-regulation in nucleus. It showed that light exposure to retina elevated Nrf2 translocation into the nucleus of the ONL cells. A further increase in Nrf2 nuclear accumulation occurred in the alpha-mangostin-treated retina with light exposure (Fig. 4).

### *In vitro*

#### Alpha-mangostin protected ARPE-19 cells against H_2_O_2_-induced cytotoxicity

**Figure 5:**
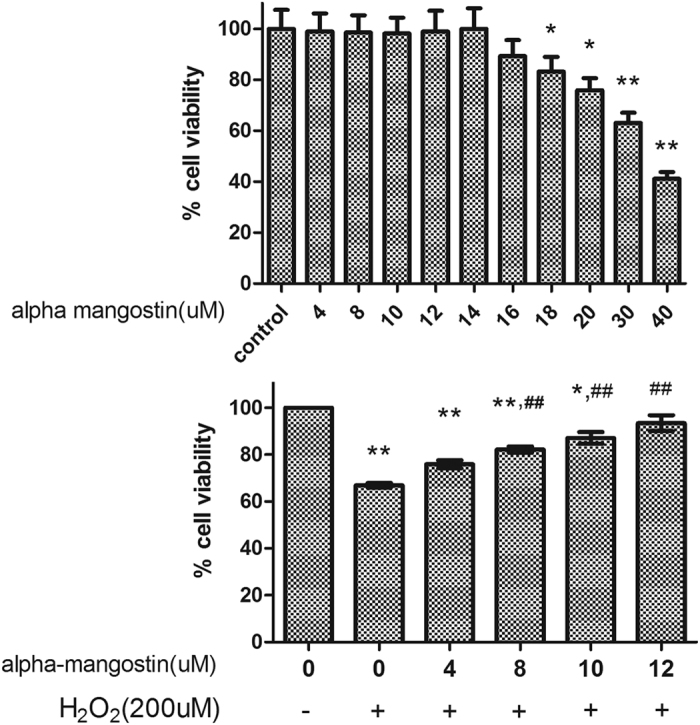
Effects of alpha-mangostin on ARPE-19 cell viability. (**A**) ARPE-19 cells were treated with indicated concentrations of alpha-mangostin (0, 4, 8, 10, 12, 14, 16, 18, 20, 30, 40 μM) for 24 hours and cell viability was measured by CCK8 assay. Results showed that: alpha-mangostin within 0–14 μM is safe to cells’ survival in ARPE-19 cells. n = 5, *P < 0.05, **P < 0.01 vs. the control cells. (**B**) ARPE-19 cells were pretreated with the indicated concentrations of alpha-mangostin (0, 4, 8, 10, 12 μM) for 24 hours, followed by H_2_O_2_ (200 μM) administration for another 24 hours. Then cell viability was tested. Alpha-mangostin inhibits H_2_O_2_-induced cell apoptosis with 0–12 μM. Significant differences were calculated using one-way ANOVA followed by Tukey’s multiple comparison test. n = 5, *P < 0.05, **P < 0.01 vs. the control cells, ^#^P < 0.05, ^##^P < 0.01 vs. the H_2_O_2_-stressed cells. Experiments were repeated three times, and similar results were obtained.

According to our experiments (Fig. 5A), alpha-mangostin up to 12 μM was selected. And 200 μM H_2_O_2_ was used in following experiments, which induced an approximate 40% loss of cell viability. Results of CCK-8 assay in Fig. 5B showed that the protective effect of alpha-mangostin against 200 μM H_2_O_2_ was significant at 4–12 μM in a concentration-dependent way, and the cell viability of alpha-mangostin at two concentrations measured by the CCK-8 was 75.94% (with 4 μM alpha-mangostin) and 93.47% (with 12 μM alpha-mangostin), respectively (Fig. 5).

#### Alpha-mangostin prevented ARPE-19 cells against H_2_O_2_–induced cell apoptosis

**Figure 6:**
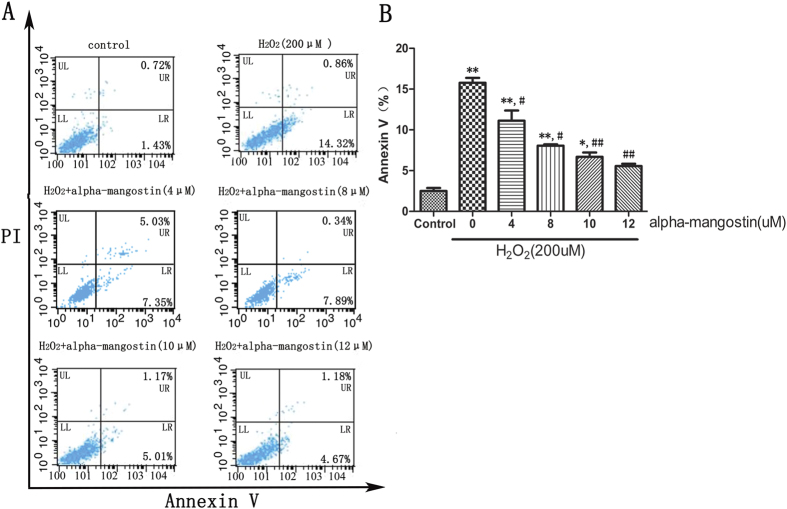
Alpha-mangostin inhibits H_2_O_2_-induced cell apoptosis in ARPE-19 cells. ARPE-19 cells were pretreated with the indicated concentrations of alpha-mangostin (0, 4, 8, 10, 12 μM) for 24 hours, followed by H_2_O_2_ (200 μM) administration for another 24 hours . Apoptosis was measured by flow cytometry using Annexin-V and propidium iodide double staining (PI). (**A**) While cells showed an increase in H_2_O_2_-induced apoptosis, there is a significant decrease in apoptosis in alpha-mangostin (4, 8, 10, 12 μM) pretreated cells. (**B**) Graphs represent the combined percentage of early (UR) and late (LR) apoptosis relative to all cells. Significant differences were calculated using one-way ANOVA followed by Tukey’s multiple comparison test. n = 5, *P < 0.05, **P < 0.01 vs. the control cells, ^#^P < 0.05, ^##^P < 0.01 vs. the H_2_O_2_-stressed cells. Experiments were repeated three times, and similar results were obtained.

ARPE-19 cells were incubated with 4 to 12 μM alpha-mangostin for 24 hours and then exposed to 200 μM H_2_O_2_ for another 24 hours. To identify whether alpha-mangostin protects against 200 μM H_2_O_2_-induced apoptosis, cells were then stained with Annexin V⁄ PI, and the apoptosis rate was determined with flow cytometry. The exposure to 200 μM H_2_O_2_ for 24 hours would lead to a significant higher rate of apoptosis (apoptotic rate: 15.18%) compared with the control cells (apoptotic rate: 2.15%). Pretreatment of alpha-mangostin could attenuate the 200 μM H_2_O_2_-induced apoptosis. High concentration (12 μM) of alpha-mangostin would lead to a more significant effect (apoptotic rate: 5.85%) compared with the lower concentration (4 μM) (apoptotic rate: 9.88%, P < 0.05) (Fig. 6).

#### Alpha-mangostin decreases H_2_O_2_-induced ROS productions in ARPE19

**Figure 7:**
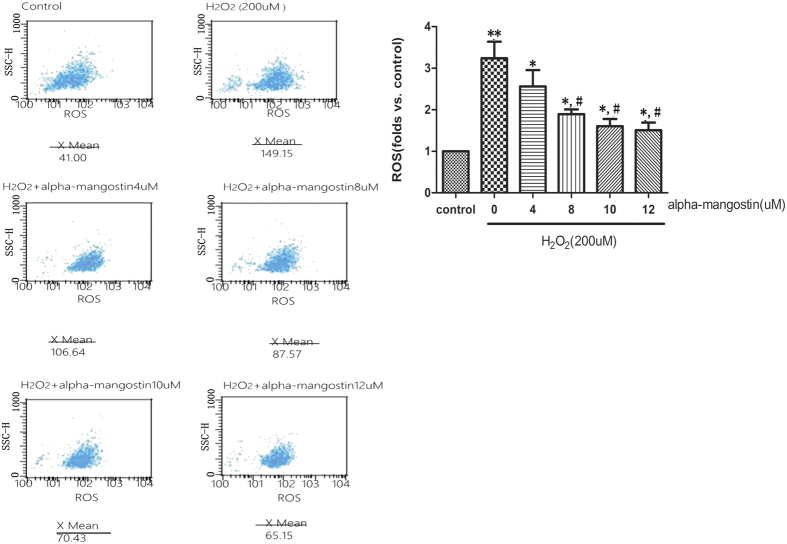
Alpha-mangostin inhibits H_2_O_2_-induced ROS production in ARPE-19 cells. ARPE-19 cells were pretreated with the indicated concentrations of alpha-mangostin (0, 4, 8, 10, 12 μM) for 24 hours, followed by H_2_O_2_ (200 μM) administration for another 24 hours. The expression of ROS was detected by Flow cytometry assay. (**A**) X-axis stands for the DCFH spots which represented the ROS production. Y-axis stands for the cell numbers. X Mean was the average ROS production. (**B**) Graphs represent the folds relationship of ROS production in all cells compared with control group. Significant differences were calculated using one-way ANOVA followed by Tukey’s multiple comparison test. n = 5, *P < 0.05, **P < 0.01 vs. the control cells, ^#^P < 0.05, ^##^P < 0.01 vs. the H_2_O_2_-stressed cells. Experiments were repeated three times, and similar results were obtained.

Excessive ROS production is one of the hallmarks of oxidative stress. To determine the effect of alpha-mangostin (0–12 μM) on the intracellular production of ROS, DCF fluorescence was recorded by flow cytometry. It revealed that mean fluorescence (i.e., intracellular ROS production) increased significantly in the 200 μM H_2_O_2_-exposed cells. In addition, treatment of the cells with alpha-mangsotin inhibited intracellular production of ROS in a concentration-dependent way which was induced by 200 μM H_2_O_2_ (Fig. 7).

### Same Experimental Sections

#### Alpha-mangostin decreased caspase-3 activity *in vitro* and *in vivo*

**Figure 8:**
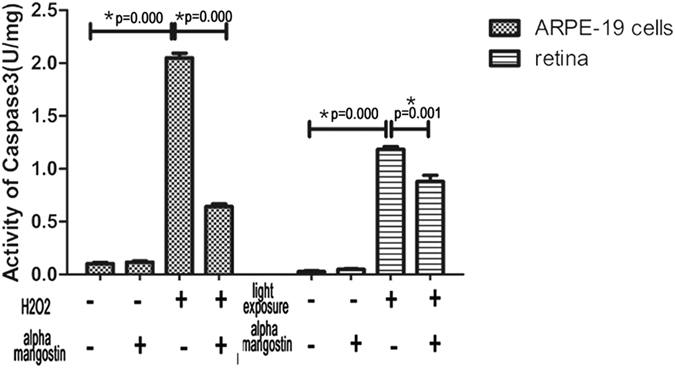
Effects of alpha-mangostin on caspase-3 activity *in vivo* and *in vitro*. Quantification analysis of caspase-3 activity both in cultured ARPE-19 cells and light-damage retina. Data are expressed as the mean ± SD. Significant differences were calculated using one-way ANOVA followed by Tukey’s multiple comparison test. n = 5, *P < 0.05. Experiments were repeated three times, and similar results were obtained.

The level of apoptic markers caspase-3 was markedly increased in 200 μM H_2_O_2_-stressed cells (2.05 ± 0.075 U/mg protein). However, alpha-mangostin significantly prevented caspase-3 activation induced by 200 μM H_2_O_2_ (0.64 ± 0.045 U/mg protein, P < 0.05). In retina tissue, caspase-3 was activated by light exposure (1.19 ± 0.043 U/mg protein). Alpha-mangostin had the same effect on caspase-3 activity in model of retinal light damage (0.88 ± 0.10 U/mg protein, P < 0.05). It did not affect normal cells or retina in caspase-3 activity (P > 0.05) (Fig. 8).

#### Alpha-mangostin suppressed the generation of lipid peroxides *in vitro* and *in vivo*

ARPE-19 cells and retinal lipid peroxidation were assessed at 24 hours after damage model, respectively. According to above results, there is no significant difference between the protective effect of 10 μM and 12 μM alpha-mangostin on RPE cells (P > 0.05). Therefore, alpha-mangostin at 10 uM was decided in this experiment. A remarkable increase of MDA level was observed in ARPE-19 cells treated with 200 μM H_2_O_2_ 24 hours (4.22 ± 0.028 nmol/mg protein), which was suppressed by pretreatment with 10 μM alpha-mangostin (1.74 ± 0.57 nmol/mg protein, P < 0.05).

**Figure 9:**
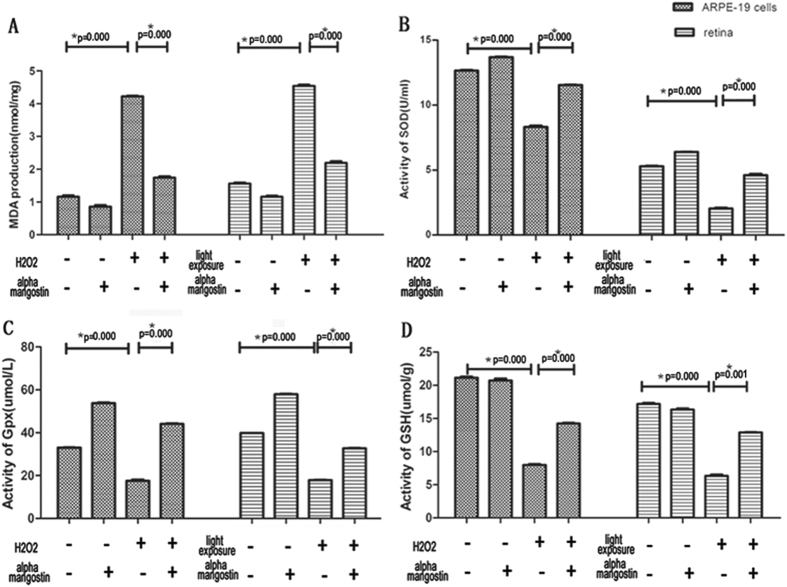
Effects of alpha-mangostin on the expression of MDA, SOD, Gpx and GSH levels *in vivo* and *in vitro*. (**A**) Quantification analysis of concentration of MDA production both in cultured ARPE-19 cells and light-damage retina. (**B**) Quantification analysis of SOD activity both in cultured ARPE-19 cells and light-damage retina. (**C**) Quantification analysis of GPX activity both in cultured ARPE-19 cells and light-damage retina. (**D**) Quantification analysis of concentration of GSH both in cultured ARPE-19 cells and light-damage retina. Data are expressed as the mean ± SD. Significant differences were calculated using one-way ANOVA followed by Tukey’s multiple comparison test, n = 5, *P < 0.05. Experiments were repeated three times, and similar results were obtained.

In addition, light exposure to retina produced excessive lipid peroxides (4.5 ± 0.57 nmol/mg protein). Daily administration of alpha-mangostin (30 mg/kg) significantly blocked the light-induced generation of MDA (2.19 ± 0.070 nmol/mg protein, P < 0.05). Alpha-mangostin alone (1.16 ± 0.045 nmol/mg protein) reduced the level of MDA to a small extent compared with normal mice (1.56 ± 0.049 nmol/mg protein, P > 0.05) (Fig. 9A). In conclusion, alpha-mangostin protected both RPE cells and retina against oxidative stress.

#### Alpha-mangostin decreased the consumption of antioxidant biomolecules *in vitro* and *in vivo*

Superoxide dismutase (SOD) activity: The SOD activity of ARPE-19 cells treated with 200 μM H_2_O_2_ for 24 hours decreased significantly (8.32 ± 0.14 U/mg protein) when compared to control cells (12.66 ± 0.071 U/mg protein, P < 0.05). Pretreatment with alpha-mangostin (12 μM) before 200 μM H_2_O_2_-stressed significantly increased the activity of SOD (11.45 ± 0.057 U/mg protein, P < 0.05).

The activity of SOD in light-exposed retina (2.03 ± 0.11 U/mg protein) reduced dramaticallyversus the normal mice (5.33 ± 0.042 U/mg protein, P < 0.05). Alpha-mangostin (30 mg/kg) supplement suppressed the reduction induced by light exposure (4.55 ± 0.14 U/mg protein, P < 0.05). Without 200 μM H_2_O_2_ and light-induced damage, SOD activity in RPE cells (13.69 ± 0.057 U/mg protein) and retina tissue (6.38 ± 0.042 U/mg protein) increased slightly (p > 0.05) (Fig. 9B).

Glutathione peroxidase (Gpx) activity: The activity of Gpx in ARPE-19 cells treated with H_2_O_2_ for 24 hours decreased significantly (17.58 ± 0.53 U/mg protein) when compared to control cells (32.94 ± 0.15 U/mg protein, P < 0.05) and cells treated with alpha-mangostin before exposed to 200 μM H_2_O_2_ (44.13 ± 0.22 U/mg protein, P < 0.05).

The activity of Gpx in light-exposed retina reduced remarkably (17.89 ± 0.21 U/mg protein) versus the normal mice (39.82 ± 0.06 U/mg protein, P < 0.05). Alpha-mangostin (30 mg/kg) supplement suppressed the reduction induced by light exposure (32.72 ± 0.36 U/mg protein, P < 0.05). It should be noted that treatment with alpha-mangostin alone markedly increased the activity of Gpx both in RPE cells (53.79 ± 0.28 U/mg protein, P < 0.05) and retina (57.90 ± 0.39 U/mg protein, P < 0.05) (Fig. 9C).

Concentration of Glutathione (GSH): GSH level of RPE cells treated with 200 μM H_2_O_2_ decreased significantly (7.99 ± 0.21 umol/g protein) when compared to control cells (21.41 ± 0.32 umol/g protein, P < 0.05) and cells treated with alpha-mangostin before exposed to 200 μM H_2_O_2_ (14.23 ± 0.20, P < 0.05umol/g protein, P < 0.05).

After light exposure, the concentration of GSH in alpha-mangostin-treated (30 mg/kg) mice increased (12.89 ± 0.12umol/g protein) when compared to vehicle-treated mice (6.35 ± 0.36umol/g protein, P < 0.05). It showed that alpha-mangostin significantly reduced GSH depletion induced by 200 μM H_2_O_2_ in ARPE-19 cells and by light damage in mice retina (Fig. 9D).

#### Upregulation of the Nrf2 and HO-1 in alpha-mangostin-induced cytoprotection *in vitro* and *in vivo*

**Figure 10:**
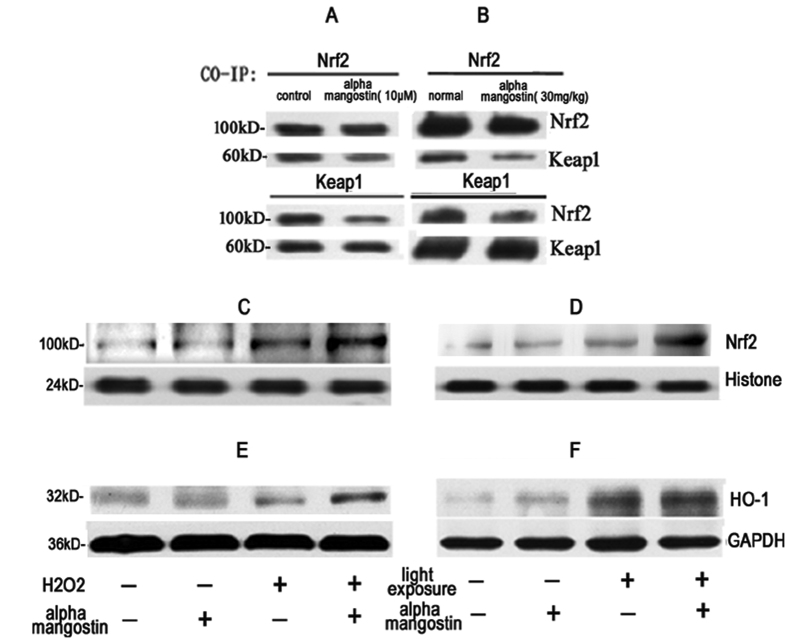
Alpha-mangostin activates Nrf2/HO-1 *in vitro* (**A**,**C**,**E**) and *in vivo* (**B**,**D**,**F**). (**A**) ARPE-19 cells and (**B**) mice were treated with alpha-mangostin respectively, and the association between Keap1 and Nrf2 was examined by co-IP. (**C**–**D**) Nrf2 expression in nucleus and (E-F) HO-1 protein expression were assayed by Western blot. GAPDH was used as internal control in total protein extract (E and F), and Histone was used as internal control in nuclear protein extract (**C**,**D**).

Nrf2 is an important transcription factor for regulating the expression of endogenous antioxidant enzymes, including heme oxygenase-1(HO-1),when it was activated and dissociated from Kelch-like ECH-associated protein-1(Keap-1).And our results above showed that alpha-mangostin prevented oxidative stress both in retina tissue and RPE cells, so we examined HO-1, Keap1, Nrf2 both in retina tissue and RPE cells to determine whether the Nrf2 and HO-1 are involved in the protective effect of alpha-mangostin against oxidative stress. The results from CO-IP assay showed that 10μM alpha-mangostin stimulation induced the disassociation between Nrf2 and Keap1 in cytosol(Fig10A).*In vivo*, we also observed that alpha-mangostin at the dose of 30mg/kg body weight dissociated Nrf2 from Keap1(Fig. 10B).The results of western blot indicated that treatment with alpha-mangostin significantly increased the expression of Nrf2 in nuclear both *in vitro*(Fig. 10C) and *in vivo*(Fig. 10D, p < 0.05).Thus, we confirmed that alpha-mangostin could dissociate Nrf2 from keap1 and help Nrf2 transfer to nuclear, which induce Nrf2 protein accumulation and nuclear translocation.

Moreover, results in Fig. 10 (C,D) showed Nrf2 expression in nuclei protein was up-regulated after light exposure, and pretreatment with alpha-mangostin induced a higher increase in the expression of Nrf2. Results in Fig. 10 (E,F) also suggested that the expression of HO-1 increased after light exposure. The expression of HO-1 in alpha-mangostin-treated mice with light exposure was significantly higher than that in the vehcile mice with light exposure. Furthermore we confirmed that alpha-mangostin induced an increase expression of Nrf2 and HO-1 in response to 200 μM H_2_O_2_ in ARPE-19 cells. These results suggested that the Nrf2/HO-1 pathway may have a key role in alpha-mangostin-induced cytoprotection against oxidative stress.

#### Alpha-mangostin induces PKC-δ protein expression *in vivo* and *in vitro*

**Figure 11:**
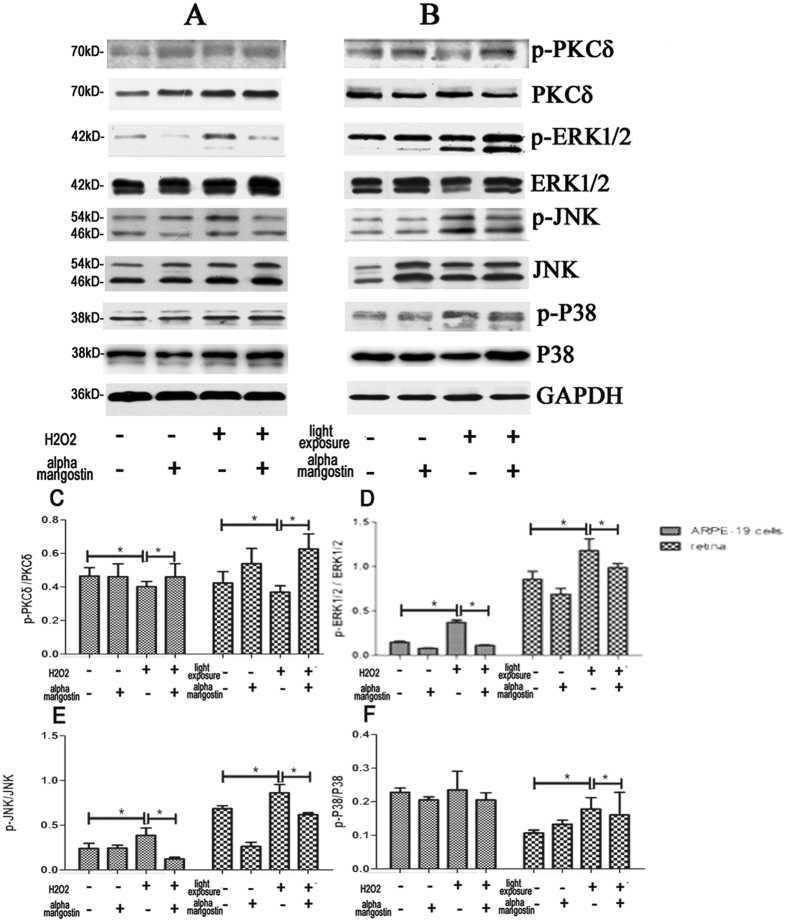
Effects of alpha-mangostin on the protein expression of PKC-δ and MAPKs (ERK1/2, JNK, P38) levels *in vivo* (**B**) and *vitro* (**A**) by Western blot. GAPDH was used as internal control. (**C**) The ratio of phospho-PKC-δ to PKC-δ was determined by densitometry. (**D**) The ratio of phospho-ERK1/2 to ERK1/2 was determined by densitometry. (**E**) The ratio of phospho-JNK to JNK was determined by densitometry. (**F**) The ratio of phospho-P38 to P38 was determined by densitometry. Data are expressed as the mean ± SD. n = 5. Significant differences were calculated using one-way ANOVA followed by Tukey’s multiple comparison test, *P < 0.05.

**P**KC-δ is currently accepted to be a kinases which can activate Nrf2^23^. It can cause Nrf2 to release from Keap1 and transfer to the nucleus. Western blot has been tested to determine the changes of PKC-δ expression in the process of alpha-mangostin-induced protective effect against oxidative stress. As showned in Fig. 11, oxidative stress significantly inhibited PKC-δ phosphorylation both *in vivo* and *vitro.* However, PKC-δ phosphorylation was restoring after the treatment of alpha-mangostin. These results revealed the involvement of PKC-δ in the protective effect of alpha-mangostin on oxidative stress-induced cell apoptosis both *in vivo* and *vitro* models.

#### Alpha-mangostin inhibits mitogen-activated protein kinases (MAPKs) activation *in vivo* and *in vitro*

It was reported that ROS generation could trigger the activation of the MAPKs including ERK1/2, p38, and JNK^24^. The Western blot results in Fig. 11 demonstrated that ERK1/2 and JNK were significantly activated (ERK1/2 and JNK phosphorylation) both in 200 μM H_2_O_2_-stressed ARPE-19 cells and light-exposed retina, which was largely inhibited by pretreatment with alpha-mangostin. We demonstrated that in mice model, treatment with alpha-mangostin suppressed p38 phosphorylation which was induced by light damage. However, there was no significant p38 phosphorylation to be observed in 200 μM H_2_O_2_-stressed ARPE-19 cells. Thus, these data revealed that the effect of MAPK inhibition may have a link with the protective effect of alpha-mangostin against oxidative stress *in vivo* and *in vitro*.

## Discussion

Alpha-mangostin has protective effect on kidney^25^, heart^18^ and brain tissues^26^ against oxidative stress, but its effects on retina have not been reported. In this study, we observed that administration with alpha-mangostin provided structural and functional protection of photoreceptor cells against light damage, which also protected ARPE-19 cells from oxidative stress-induced damage. Antioxidant status was modulated by alpha-mangostin including increase of SOD, Gpx activities and GSH content. We also found that up-regulation of the expression of phase II enzyme such as HO-1 was accompanied by the activation of Nrf2 after supplementation with alpha-mangostin. These results suggested that alpha-mangostin may protect retina against oxidative stress through up-regulating Nrf2 activity.

In the light damage model, a reduction in the ONL thickness suggested that light exposure resulted in photoreceptor degeneration, which was in correlation with the decrease of dark adapted a-wave amplitude. Pretreatment with alpha-mangostin preserved photoreceptor cells, which lead to protection of retinal function. The alterations occur in the RPE, following the changes in the photoreceptor outer segments after light damage. Laboratory evidence has demonstrated that photochemical reactions caused by visible light in the oxygen-rich environment of the outer retina lead to the liberation of cytotoxic ROS. These ROS cause oxidative stress, which is one of the major factors of onset and progression of the death of RPE^27^,^28^. Our results *in vitro* showed that alpha-mangostin also inhibited H_2_O_2_-mediated RPE cell viability loss at the concentration of up to 12 μM. The apoptosis of retinal cells as a main pathway in response to retinal light damage is operated by diverse mechanisms^29^. Caspase-3 is the terminal effector of the apoptotic machinery and its activation is a sign of irreversible phase of apoptosis^30^. In this study, an increase in the number of apoptotic cells was observed after light exposure in the outer nuclear layer. And treatment with alpha-mangostin remarkably inhibited photoreceptor apoptosis and caspase-3 activation induced by light damage in retina. We also found the effective anti-apoptotic activity of alpha-mangostin through modulating caspase pathway at lower dosage in response to oxidative stress *in vitro*.

Oxidative stress is known to be crucial in the process of light-induced retinal cell death^31^. In our study, the level of oxidative damage induced by light exposure was measured by TBARS. We observed MDA, a bio-marker of lipid peroxidation, increased significantly in light-exposed retina tissues as well as 200 μM H_2_O_2_-stressed ARPE-19 cells. Pretreatment of alpha-mangostin diminished this MDA production both *in vivo* and *in vitro*, which suggested that alpha-mangostin with antioxidant activity protected retinal cells from light damage via reducing oxidative stress. To underlie the mechanism of alpha-mangostin in response to oxidative stress, we examined the level of ROS in ARPE-19 cells stressed by 200 μM H_2_O_2_ firstly. The results showed that alpha-mangostin scavenged intracellular ROS *in vitro* model. Intensive light exposure produces excessive ROS and makes antioxidants consumption, which disrupt the balance between pro-oxidant and anti-oxidants and lead to oxidative stress^32^. Two modes of antioxidant action are available in tissues: 1) direct scavenge of free radicals and 2) generation of antioxidant bio-molecules, both processes act to reduce the level of oxidative stress^33^. Then we demonstrated that administration with alpha-mangostin leaded to a significant recovery of endogenous antioxidant defense system including the activity of SOD, GPX, and content of GSH both *in vitro* and *in vivo* model. In addition, activity of GPX was up-regulated by alpha-mangostin alone. We assumed that pretreatment alpha-mangostin both enhanced GPX activity and scavenged ROS directly to reduce depletion of GSH and to inhibit lipid peroxidation, as evidenced in earlier reports.

HO-1 is recognized as a cytoprotective defense system and an inducible enzyme in response to oxidative stress^34^. Our observations suggested that besides an increase in HO-1 protein expression mediated by 200 μM H_2_O_2_ in ARPE-19 cells, an endogenous defense system with increasing HO-1 expression was also initiated by light exposure in retina. Furthermore, it has been reported that neuroprotective effects of alpha-mangostin was partially mediated by the induction of HO-1, which is heme-degradation enzyme and rate-limiting enzyme can catalyze the degradation of heme to carbon monoxide, catalytic iron and bilirubin^34^. Echoing with previous researches, our data showed a further increase of HO-1 protein expression was induced by pretreatment with alpha-mangostin both *in vivo* and *in vitro* model, which suggested that HO-1 was involved in the protective effects of alpha-mangostin against oxidative stress-induced cell death.

To further investigate the protetcive effect of alpha-mangostin, we examined the nuclear factor erythroid 2-related factor 2 (Nrf2) protein, which is a transcription factor that plays a central role in protective molecular response to oxidative stress in most cells, including RPE cell^35^. Under the stimulus of antioxidant and oxidative stress, Nrf2 activated and dissociated from Keap1. The activated Nrf2 translocated into the nucleus and interacts with antioxidant response elements (AREs), which mediated transcriptional induction of various antioxidants, including SOD, GPX and HO-1. It was reported that generation of ROS could trigger Nrf2 activation^36^. Our data showed that light exposure and 200 μM H_2_O_2_ up-regulated the protein expression of Nrf2 in nuclear, which implied Nrf2 was involved in the endogenous defense system. Pretreatment of alpha-mangostin induced a higher level of Nrf2 expression in nuclear. Furthermore, we confirmed that alpha-mangostin both at the dose of 30 mg/kg body weight in retina tissue and at the concentration of 10 μM in ARPE-19 cells disrupted the binding of Nrf2 with Keap1, promoted Nrf2 nuclear translocation by co-immunoprecipitation. Concurrent with a further increased expression of Nrf2/HO-1 stimulated by alpha-mangostin, the apoptosis of photoreceptor cells, dysfunction of retina were also significantly inhibited by pretreatment of alpha-mangostin in light damage model. Combined the same effects of alpha-mangostin we observed *in vitro* model, we speculated that protective effects of alpha-mangostin against oxidative stress were mediated via the activation of Nrf2/HO-1 pathway. The limitation of our study is that more evidences of activation of Nrf2/HO-1 pathway in the process of alpha-mangostin in response to light-induced cell death are needed in the future studies.

Nrf2 could be modulated by some signaling kinases, such as PKC kinases^37^, MAPKs (ERK1/2, p38, JNK) . PKC kinases induce Nrf2 phosphosphorylation, which is established as a regulator and upstream signal for Nrf2 activation. Furthermore, PKC inhibition blocks Nrf2 phosphorylation and transcriptional activity^38^. We observed PKC-δ phosphorylation after treated with alpha-mangostin. We speculated that PKC-δ participated in the modulation of Nrf2 transcriptional activity in the process of alpha-mangostin protecting retinal cells against oxidative stress. Additionally, studies showed that MAPK is involved in cell survival against oxidative stress through the Nrf2 signaling pathway^39^,^40^. Conversely, alpha-mangostin attenuated 200 μM H_2_O_2_ and light-induced JNK, p38, and ERK1/2 activation, implying that alpha-mangostin may activate Nrf2 in a MAPK-independent manner. More investigations are needed to determine the role of PKC-δ in the modulation of Nrf2 transcriptional activity in the process of alpha-mangostin protecting retinal cells against oxidative stress and whether protective effect of alpha-mangostin against 200 μM H_2_O_2_ and light damage is associated with MAPKs inhibition.

Importantly, our results provided evidences that alpha-mangostin with intragastric administration crossed the blood-retina barrier and further accumulated in the retina after multiple doses. More experiments are needed to establish the concentration of alpha-mangostin in retina after intragastric administration.

In conclusion, we demonstrate that alpha-mangostin can suppress retinal cells apoptosis induced by oxidative stress by scavenging ROS and strengthening the endogenous defense system. Alpha-mangostin can protect retina and RPE cells against oxidative stress via enhancing Nrf2 transcriptional activity, translocating Nrf2 to nucleus and increasing the expression of HO-1. It also can modulate the expression of PKC-δ and MAPKs. It indicated that alpha-mangostin could be a new approach to suspend the onset and development of AMD.

## Materials and Methods

### Regents and Materials

Alpha-mangistin powder (Product M3824, purity >98%), hydrogen peroxide (H_2_O_2_) (Product H1009, 30% (w/w) in H_2_O), and 4’, 6-Diamidino-2-phenylindole (DAPI, Product D9542) were supplied by Sigma (St. Louis, MO, USA). Cell culture supplies were obtained from Hyclone (Logan, Utah, USA). Rabbit polyclonal anti-Nrf2 antibody (ab137550) purchased from Abcam (Cambridge, MA, USA) and Alexa Fluor 488 of goat anti-rabbit immunoglobulin G (IgG) (H + L) got from Invitrogen (A11070, Carlsbad, CA, U.S.A.) were used in immunofluorescence. The Nrf2, HO-1 and Keap-1 antibodies were purchased from Cell Signaling Technology (Danvers, MA, USA). Anti-ERK1/2, anti-p-ERK1/2, Anti-JNK, anti-p-JNK, Anti-P38, anti-p-P38, Anti-PKC-δ, anti-p-PKC-δ, used in western blot were obtained from Santa Cruz Biotechnology (Santa Cruz, CA, USA).

### *In vivo*

#### Mice treatment

Mice choosing: Female balb/c mice, aged 8 weeks, were obtained from the Experimental Animal Center of Nanjing Medical University. They were raised in normal cages in a 12:12-h light–dark cycle with food and water ad libitum. All experiments were performed in accordance with the Association for Research in Vision and Ophthalmology (ARVO) Statement for the Use of Animals in Ophthalmic and Vision Research, and they were approved and monitored by the Institutional Animal Care and Use Committee of Nanjing Medical University.

Treatment with Alpha-mangostin and Exposure to white light: Alpha–mangostin was dissolved in corn oil. According to the studies of alpha-mangostin on other tissues^41^, the mice with alpha-mangostin treatment were treated with the dose of 10 mg/kg (lower-dose) or 30 mg/kg body weight (higher-dose) and the vehicle mice were treated with corn oil by intragastric administration daily for 7 days. Equivalent volumes of vehicle were treated to the vehicle mice. Normal mice did not receive any treatment. On the fifth day after the administration, mice were exposed to white light as the following. After dark adaption for 24 hours, the pupils were dilated with a mixture of 0.5% tropicamiden and 0.5% phenylephrine (Mydrin-P; Santen, Osaka, Japan) at 30 mins in dim red light before light exposure. Unrestrained mice were exposed to the surrounding 5000lux white light-emitting diodes (LEDs) for 1 hour (9 AM to 10 AM everyday). After exposure to white light, mice were placed in darkness with food and water at will for additional 24 hours, then they were returned to the normal housing conditions until the examination.

Retinal sections: Mice were sacrificed after the last ERG recording, and both eyes were surgically collected. Paraffin-embedded retinal sections: the eyeballs were fixed in 4% paraformaldehyde for 24 hours. Then, the eyeballs were immersed in 55, 65, 75, 85 and 95% (v/v) ethanol for 30 mins successively for dehydration. Subsequently, Eyes enucleated were placed in 100% (v/v) ethanol twice at room temperature for 30 mins. The eyecups were infiltrated in chloroform for 5 mins before embedded in paraffin. Sections (5 μM thick) of the entire retina, including the optic nerve, were cut along the vertical meridian of each eyeball and stained with hematoxylin and eosin (H&E).

Frozen retinal sections: The eyes were extracted and sent to O.C.T. Compound (SAKURA) for frozen sections right away, kept at −80 °C. Retinal sections were cut at 8μm thickness at −20 °C and stored at −80 °C until staining.

Protein extraction from retina: After treatments, mice were euthanized, and the eyeballs were removed. The retinas were separated from the eyeballs. Mice retinas were homogenated with ice-cold RIPA Lysis Buffer (Beyotime, P0013, China) mixed with phosphatase inhibitors, protease inhibitor and phenylmethyl sulfonyl fluoride (PMSF). The homogenate was incubated at 4 °C for 30 min and centrifuged at 12,000g for 20 min at 4 °C. The supernatant was obtained as total protein extract.

The nuclear protein and cytoplasmic protein were isolated using a Nuclear and Cytoplasmic Protein Extraction Kit (Beyotime, P0028, China) based on the instructions provided. Protein concentrations were determined with a BCA protein assay kit (Beyotime, Shanghai, China).

#### Experimental Section

Electroretinogram: Full-field Electroretinogram (ERG) was performed 3 days after light exposure. Mice were maintained in complete darkness for 24 hours. Mice were intraperitoneally anesthetized with a mixed solution of ketamine (120 mg/kg body weight) and xylazine (6 mg/kg body weight). The mydriatic drop was applied to dilate the pupils as previously mentioned. ERGs were recorded in left eye of the dark-adapted mice by placing gold wire electrodes centrally on the cornea, which were protected by viscoelastic. And a reference electrode was placed through the cheeks. A ground electrode was inserted subcutaneously near the tail. All procedures were completed in dim red light. For the evaluation of rod photoreceptor function (scotopic ERG), five strobe flash stimuli were presented in a Ganzfeld with flash intensities at 0.0095 cds/m2 (−25 dB), 0.095 cds/m2 (−15 dB), 0.95 cds/m2 (−5 dB), 3 cds/m2 (0 dB) and 9.49 cds/m2 (5 dB). The amplitude of the a-wave was measured from the baseline to the maximum a-wave peak, and the b-wave was measured from the trough of the a-wave to the peak of the b-wave. The a-wave shows the function of the photoreceptors, while the b-wave reflects the function of bipolar cell and Müller cell.

Histological analysis of paraffin-embedded retinal sections: Mice were sacrificed after the last ERG recording, and both eyes were surgically collected. The eyeballs were fixed in 4% paraformaldehyde for 24 hours. Then, the eyeballs were immersed in 55, 65, 75, 85 and 95% (v/v) ethanol for 30 mins successively for dehydration. Subsequently, Eyes enucleated were placed in 100% (v/v) ethanol twice at room temperature for 30 mins. The eyecups were infiltrated in chloroform for 5 mins before embedded in paraffin. Sections (5 μm thick) of the entire retina, including the optic nerve, were cut along the vertical meridian of each eyeball and stained with hematoxylin and eosin (H&E). The thickness of the outer nuclear layer (ONL) was measured at 240-μm intervals starting next to the optic disc along the superior and inferior hemiretina. Data from six sections were averaged for each eye.

TUNEL staining of Paraffin-embedded retinal sections: Eyeballs of mice were harvested 24 hours after light exposure. Paraffin-embedded retinal sections were made as previously mentioned in this experiment. Transferase-mediated deoxyuridine triphosphate-biotin nick endlabeling (TUNEL) staining was performed according to the manufacturer’s protocols (*In Situ* Cell Death Detection kit; Roche Biochemicals, Mannheim, Germany) to detect the apoptotic cells. Nuclei were stained with DAPI (Sigma). Fluorescence images were photographed, and TUNEL-positive cells in the outer nuclear layer at a distance between 240 and 720 μM from the optic disc were obtained in the superior area of the retina. The intensity of TUNEL-positive cells was averaged for these superior areas.

Immunofluorescence for Nrf2 of frozen retinal sections: The frozen slices were washed with phosphate-buffered saline (PBS) and blocked with bovine serum albumin (BSA) solution at 37 °C for 1h. Then the sections were incubated overnight in rabbit polyclonal anti-Nrf2 antibody (Abcam) diluted 1: 200 at 4 °C. Then the slices were washed with PBS three times, a mixture of an Alexa Fluor 488 of goat anti-rabbit immunoglobulin G (IgG) (H+ L) (1: 1000 dilution) was applied as secondary antibodies and then incubated at 37 °C for 2 hours. The slices were washed again with PBS three times (5 mins/time). Cell nuclei were counterstained with 4’, 6-diamidino-2-phenylindole (DAPI).We confirmed the staining by comparing with the negative control. The sections were analyzed using fluorescence microscopy with identical exposure parameters.

Caspase-3 activity measurement of total retinal protein extraction: Caspase-3 is an intracellular cysteine protease that exists as a pro-enzyme, playing a key role in the process of caspase-dependent apoptosis. Total retinal protein extraction was harvested for the assay. Caspase-3 activity was detected with a Caspase 3 Colorimetric Assay Kit (BioVision) according to the manufacturer’s instructions. Read the samples at 405nm in a microtiter plate reader. Caspase-3 activity was calculated according to the light absorption value of the samples and negative control relative absorption ratio value.

MDA detection of total retinal protein extraction: The level of malondialdehyde (MDA), which is an end-product of lipid peroxidation and a biomarker of cellular oxidative stress, was determined by OxiSelect™ TBARS Assay Kit (Cell BioLabs) according to the manufacturer’s instructions. Total retinal protein extraction was included in the experiment.

SOD, GPX and GSH levels of total retinal protein extraction: Enzyme activities (IU/mg protein) in total retinal protein extraction were measured . Estimations of antioxidant enzymes such as Superoxide Dismutase (SOD) and Glutathione Peroxidase (GPX) were performed by Superoxide Dismutase (SOD) Activity Assay Kit and Glutathione Peroxidase Activity Colorimetric Assay Kit from Bio Vision, Inc (San Francisco, USA) , as per manufacturer’s instructions, respectively. The level of glutathione (GSH) was estimated using Glutathione Colorimetric Assay Kit (Bio Vision). The absorbance of each sample was read at 405 nm. The concentrations of GSH in the samples were calculated according to the standard glutathione calibration curve.

Coimmunoprecipitation (co-IP) assay: We did co-IP assay to demonstrate that alpha-mangostin activated Nrf2 and dissociated Nrf2 from Keap1.As previously described, after treatment, retina was homogenated with ice-cold RIPA Lysis Buffer (Beyotime, P0013, China) containing 0.3% Chaps to preserve the integrity of the complexes. To the cleared lysates, 2μg of Keap1 antibody was added per 1.2 mg of soluble proteins, and gently rotated at 4 °C overnight. The immunocomplex was captured by adding 25 μl protein A+G agarose beads (Beyotime, Beijing, China) and gently rotating at 4 °C for 3 hours. Then the mixture was centrifuged at 2500g for 5 minutes at 4 °C and the supernatant was discarded. The precipitate was washed for three times with ice-cold RIPA buffer containing Chaps, resuspended in 3×sample buffer and boiled for 5 minutes to dissociate the immunocomplex from the beads. The supernatant was collected by centrifugation and analyzed by Western blot as described.

Western blot for Nrf2, HO-1, ERK1/2, JNK, P38 and PKC-δ: Total and phosphorylated mitogen-activated protein kinases (MAPKs), including ERK1/2, JNK, P-38, and PKC-δ levels were determined in total retinal protein extraction by western blot, as well as protein levels of HO-1. And Nrf2 was analyzed in nuclear protein extract by western blot. Total protein and nuclear protein samples were separated by 7.5–13% SDS-PAGE and then transferred onto a polyvinylidene difluoride (PVDF) membrane (Millipore, IPVH00010, Bedford, MA, USA). After being blocked with 5% (v/v) skim milk for 120 mins at room temperature, membranes were incubated with anti-Nrf2 (1:1000), anti-HO-1 (1:1000), anti-Erk1/2 (1:1000), anti-p-Erk1/2 (1:1000), anti-JNK (1:500),anti-p-JNK(1:500), anti-p38 (1:1000), anti-p-p38 (1:1000), anti-PKC-δ (1:1000), anti-p-PKC-δ (1:1000), overnight at 4 °C and washed with TBST (15 mins each time,3 times). Then the corresponding horseradish peroxidase-linked secondary antibodies were added and left for 50 mins to 60 mins at room temperature followed by washing with TBST (10 mins 3 times). The Western blot results were visualized by Chemiluminescent HRP Substrate (Millipore, WBKLS0050, Bedford, MA, USA). The intensity of each blot was quantified using Image J software after normalization to corresponding loading controls.

### *In vitro*

#### Cell treatment

Cell lines and culture: ARPE-19 cells (human retinal pigment epithelial cell line, ATCC CRL-2302) were cultured in Dulbecco’s Modified Eagle Medium: Nutrient Mixture F-12 (DMEM/F-12) supplemented with 10% fetal bovine serum (Hyclone), 100 U/mL penicillin , 100μg/mL streptomycin. The cells were incubated at 37 °C in a humidified environment containing 5% CO_2_. The cells were subcultured and, upon reaching 80–90% confluence, they were starved in serum-free DMEM/F12 overnight (12 hours) before further treatment.

Treatment with Alpha-mangostin and Exposure to H_2_O_2_: ARPE-19 cell cultures were treated with different concentrations of alpha-mangostin. Cell viability was evaluated, respectively. To test the cytoprotective effect of alpha-mangostin against H_2_O_2_-stressed cell death, the cells were pretreated for 24 hours with various concentrations of alpha-mangostin. The cells were then washed and stressed with 200 μM H_2_O_2_ for an additional 24 hours. The H_2_O_2_-stressed cells were incubated with H_2_O_2_ at 200 μM for 24 hours, while the control cells had no special treatment.

Protein extraction from ARPE-19 cells: After treatments, the cells were gently washed twice with ice-cold PBS. Then total protein lysates were prepared in RIPA Lysis Buffer (Beyotime, P0013, China) mixed with phosphatase inhibitors, protease inhibitor and PMSF for 30 mins on ice. Lysates were centrifuged at 16,000g for 20 mins at 4 °C. The supernatant was obtained as total protein extract.

The nuclear protein and cytoplasmic protein were isolated using a Nuclear and Cytoplasmic Protein Extraction Kit (Beyotime, P0028, China) based on the instructions provided. Protein concentrations were determined with a BCA protein assay kit (Beyotime, Shanghai, China).

#### Experimental Section

Cell viability assays: Cell viability was determined by Cell Counting Kit-8(CCK8). ARPE-19 cells (1 × 10^5^ cells/well) were seeded and grown in 96-well plates for 24hour, then treated as previously described. Before the evaluation of the growth rate of ARPE-19 cells, 100 μL of spent medium was replaced with an equal volume of fresh medium containing 10% CCK8 (WST-8, Dojindo Laboratories, Tokyo, Japan). Cells were incubated at 37 °C for 1-4 hours, and the absorbance value of liquid culture medium was finally determined at 450 nm using enzyme-labeling measuring instrument. Cell viability rate (%) = [A450 (sample)-A450 (blank) ]/[A450 (control)-A450 (blank)] *100.

Flow cytometry analysis of apoptosis: With or without pre-treatment, all anchorage-dependent cells and floating cells were collected, centrifuged, washed twice with PBS and resuspended in 1 × Binding Buffer. After adding 5 μL of AnnexinV-FITC solution and 5 μL of PI solution according to the instructions of AnnexinV-FITC Apoptosis Detection Kit (BD Biosciences), cells were incubated in the dark place for 15 minutes at room temperature. Immediately analyze the result with flow cytometry to calculate the percentage of the viable apoptotic cell.

Reactive oxygen species (ROS) detection: In brief, after treatment, ARPE-19 cells were incubated with 10 μM of DCFH-DA (Product D6883, sigma, St. Louis, MO, USA) in serum-free medium at 37 °C for 30 mins. Cells were trypsinized, washed and resuspended in phosphate-buffered saline (PBS), then sent for flow cytometry analysis. The percentage of fluorescence-positive cells was measured at an excitation wavelength of 488 nm and an emission wavelength of 525 nm.

Caspase-3 activity measurement of total protein extraction: Detection method of Caspase-3 activity in ARPE-19 cells was as same as mentioned in Experimental Section *in vitro above.*

SOD, GPX and GSH levels of total protein extraction: MDA detection of total protein extraction: Detection method in ARPE-19 cells was as same as mentioned in Experimental Section *in vitro* above.

Coimmunoprecipitation (co-IP) assay: Detection methods were as same as mentioned in Experimental Section *in vitro* above.

Total protein lysates extracted from ARPE-19 cells contained 0.3% Chaps to preserve the integrity of the complexes. Detection methods of co-IP assay were as same as mentioned in Experimental Section *in vitro* above.

Western blot for Nrf2, HO-1, ERK1/2, JNK, P38 and PKC-δ: Detection methods were as same as mentioned in Experimental Section *in vitro* above.

## Statistical analysis

The results were presented as the means ± standard error of the mean (SEM). One-way ANOVA or Student’s t test was performed to assess the statistical differences between the groups using SPSS19.0 software and Graphpad Prism 5.0. Statistical significance was set at p < 0.05.

## Additional Information

**How to cite this article**: Fang, Y. *et al.* Protective effect of alpha-mangostin against oxidative stress induced-retinal cell death. *Sci. Rep.* **6**, 21018; doi: 10.1038/srep21018 (2016).
